# 

**Published:** 1894-10-13

**Authors:** 


**Notices of Births, Deaths, and Marriages** are inserted in this column at a charge of 2s. 6d. for an announcement not exceeding four lines.

**Notice to Advertisers and Subscribers.**
All Advertisements, Orders for Copies of The Hospital, and Business Communications should be addressed to **The Publisher, NOT TO THE EDITOR,** The Hospital, 428, Strand, W.C.
The Cost of Subscription, post free, is as follows:—Per week, 2½d.; per quarter, 2s. 8d.; per half-year, 5s. 3d.; per annum, 10s. 6d. Foreign:—Per Annum, 12s. 8d.; payable, in all cases, in advance.

NOTICE TO CORRESPONDENTS.
All MS., Letters, Books for Review, and other matters intended for the Editor should be addressed The Editor, The Lodge, Porchester Square, London, W.
The Editor cannot undertake to return rejected MS., even when accompanied by stamped directed envelope.

**"Burdett's Hospital Annual," 1894.**
Fifth year of publication. 900 pp. Boards, gilt lettering. A most complete guide to British, Colonial, Indian, and American Hospitals, Dispensaries, Nursing and Convalescent Institutions, and Asylums. Price 5s. Published by The Scientific Press (Limited), 428, Strand, W.C.

**NOTICE.—The Index for Vol. XV. (ending March 31st, 1894) is on sale at the Office of this Journal, price 2½d., post free.**

### Scraps and Gleanings.

The report of the Royal Orthopœdic Hospital is a satisfactory one. An increase of 31 patients is reported.

Miss Laird has generously contributed £100 towards the furnishing of the new wing for the West Kirby Convalescent Home.

Although 73 years of age, Professor Virchow attended no less than five international congresses during his summer holidays.

An International Congress of a unique character is to be held at Bordeaux in July, 1895, the subject under discussion being "The Proper Protection of Children."

The patients of St. George's and University College Hospitals have had some of the Royal fruit sent them which the Queen's head gardener exhibited at the Crystal Palace.

The Woman's Hospital which has been established at Soo Chow, in China, is under the direction of an American from Mississippi named Dr. Anne Walter.

Monsieur Armaud Caille, who has just died at Chateaudun, has left his entire fortune to charitable institutions and hospitals. These various legacies amount to about 130,000 francs.

A Bill has just been introduced into the New Zealand House of Representatives which is framed to exclude from the Colony all undesirable emigrants, within which category it is proposed to include persons suffering from consumption.

The managers of the Bristol Industrial and Fine Art Exhibition have handed over to the General Infirmary of Bristol the sum of £750. This will help in a measure to clear off the very serious debt existing now on the accounts of the institution.

Madame Albani contributed £5 to the Jenny Lind Hospital at her recent visit to that institution. The great songstress further visited each ward, giving each patient a trifling souvenir of her interest in the welfare of this interesting hospital.

A State laboratory is in course of erection at St. Petersburg, with a view to studying and exterminating the so-called Siberian Pest. It is proposed to prepare for this purpose a vaccine, the lymph being sent from the Pasteur Institute at Paris.

The Eastern Counties Asylum for Idiots at Colchester has been presented by Sir Savile Crossley with a house at Clacton-on-Sea, which he purchased for this purpose at a cost of £3,000. This will constitute a sea-side home for the patients at the asylum.

The system has now been adopted in England of the Bertillon system of minute anthropometric measurements for the identification of criminals, in addition to Herschell's finger-print system, which has been made generally known by Mr. Francis Galton.

The Health Committee of St. Pancras Vestry, in consequence of the yearly increase of scarlet fever and diphtheria, have decided to visit all the schools in the parish as far as possible. Some of the schools, we hear, which have already been visited show serious defects.

The sub-committee appointed to consider the hospital accommodation question at York have instructed the City Surveyor to prepare plans for a temporary hospital on the lines of one viewed by them at Sandal, with certain improvements and alterations in the arrangements.

Professor Roux, of Paris, at Buda Pesth, reported at the recent Hygienic Congress that about 75 per cent. of the children in Paris hospitals who were inoculated with Behring's anti-diphtheritine were saved, while of those not inoculated 60 per cent. died, and only 40 per cent. survived.

The finances of the Bristol Royal Infirmary are, we hope, somewhat looking up, there having been an improvement in bequests. From Mr. Vaughans £4,000 altogether has been received. A legacy also of £1,000 is reported from Miss Leonard for the endowment of a bed in memory of her brother.

Lord Armstrong, it is reported by local papers, has signified his wish to provide a new hospital as an out-patients' department in connection with the Hospital for Sick Children, Moor Edge. This gift, including the site and furnishing, is in special commemoration of the late Lady Armstrong.

The annual general meeting of the Governors of the Lowestoft Hospital has just been held. The report is not of a very satisfactory character. The committee propose that a grand bazaar be held in August of next year, in consequence of the existing necessity for an increase of funds.

The Aldershot Hospital Saturday, formed through the Farnham Committee, and the kind interest of Mr. A. Mason, produced £190. The Royal Surrey County Hospital has received the substantial sum of £410 from Hospital Saturday collections in Godalming, Guildford, Farnham, and Aldershot.

Accounts of the progress of Dr. Haff Line's cholera inoculations in India are somewhat contradictory. Several soldiers, it is now reported, who were inoculated with the Doctor's virus at Lucknow were attacked, and the same proportion of them succumbing as did those on whom no precautionary measures had been practised.

A curious and unique entertainment was held at the Hospital for Women and Children at Cork. An "At Home" was given in celebration of the fifth anniversary of the dog Leo, who has done so much excellent begging work for the institution. Many cheques were sent as birthday presents to the dog, which went far to help the hospital exchequer.

Last year's report of the Sheffield General Infirmary noted that steps had been taken to place within the walls of the institution a permanent memorial of Mr. Favell's thirty-five years' service of honorary surgeon. That memorial has been completed in a portrait painted by J. J. Shannon, and presented by the Duke of Norfolk on behalf of the subscribers.

A definite proposal has recently been laid before the Estates and Property Committee of the Newcastle Corporation for the purchase of ground opposite the Jesus Hospital in City Road, on which it is proposed to erect a hospital for outdoor patients in connection with the Fleming Memorial Hospital, Moor Edge. This site and the hospital are to be provided, we believe, at the instance of a munificent benefactor of Newcastle, whose name we are not at present at liberty to mention.

### The Student of Medicine.

**APPOINTMENTS.**

H. M. Body, M.R.C.S.Eng., reappointed Medical Officer of Health to the Crediton Rural Sanitary Authority. Dr. Bourke, appointed Medical Officer for the East Flegg District of the Rollesby Union. J. T. Brown, M.B., C.M.Glasg., reappointed Medical Officer for the Trelleck District of the Monmouth Union. R. R. Brunskill, B.A., M.D., B.Ch.(Dub.), appointed Head Surgeon to the North Dispensary, Liverpool. E. Fernie, M.D.St.And., reappointed Medical Officer of Health to the Stone Local Board. F. W. Fullerton, M.B., B.S., M.R.C.S., L.R.C.P., appointed Medical Officer to the District of West Sculcoates, Hull. T. H. Galbraith, M.B., C.M.Aberd., appointed Medical Officer to the Wolverhampton Union Workhouse. G. E. G. Metcalf, L.R.C.P., L.R.C.S.Edin., reappointed Medical Officer for the Capel District of the Tonbridge Union. E. L. Pawlett, M.R.C.S., L.R.C.P., appointed Junior Assistant Medical Officer, Cornwall County Asylum, Bodmin. T. G. Prosser, M.R.C.S. Eng., reappointed Medical Officer for the Rockfield District of the Monmouth Union. Dr. Roydon, appointed Medical Officer of the Workhouse of the Rollesby Union. E. J. Smyth, M.B.Lond., B.S., appointed Resident Clinical Assistant to the St. Marylebone Infirmary, North Kensington. J. F. Taylor, M.R.C.S., L.S.A., appointed Medical Officer of the Wanstead Slip Division of the West Ham Union. H. B. Vincent, M.R.C.S. Eng., appointed Medical Officer of Health to the Corpingham Rural Sanitary Authority. H. S. Wild, M.R.C.S.Eng., L.R.C.P., appointed House Physician to Addenbrooke's Hospital, Cambridge.

**VACANCIES.**

The following vacancies are announced this week, the amount of salary in each case being given after the name of the institution:

**Resident Medical Officers.**

Ancoats Hospital, Manchester.—Resident House Surgeon, with charge of In-patients. Salary £80 per annum, with board and washing. Applications to Alex. Forrest, Honorary Secretary, by October 17th.

Corporation of Croydon.—Resident Medical Officer for the Borough Isolation Hospital for Infectious Diseases, must be fully qualified. Salary £120 per annum, with board and lodging, increasing by £20 at the end of the first year. Applications and testimonials endorsed "Resident Medical Officer," to be sent to the Medical Officer, 7, Katherine Street, Croydon, before October 17th.

Huddersfield Infirmary.—Junior House Surgeon. Salary £40 per annum, with board, lodging, and washing. Applications to Mr. J. Bate, Secretary, by October 15th.

National Hospital for Diseases of the Heart and Paralysis, Soho Square.—Resident Medical Officer; must be doubly qualified. Appointment for six months; residence in the Hospital and an honorarium of £10 10s. Applications and testimonials to Capt. F. Hanaley, Secretary, before October 13th.

Rotherham Hospital and Dispensary.—Assistant House Surgeon. No salary, but board, lodging, and washing provided. Appointment for six months. Applications and testimonials to the House Surgeon before October 18th.

St. Pancras and Northern Dispensary, 126, Euston Road, N.W.—Resident Medical Officer, doubly qualified. Salary £105 per annum, with residence and attendance; and a Physician-Accoucheur. Applications to H. Peter Bodkin, Honorary Secretary, 23, Gordon Street, Gordon Square, by October 22nd.

South Devon and East Cornwall Hospital, Plymouth.—Assistant House Surgeon. No salary, board and residence provided. Applications and testimonials to J. Walter Wilson, Honorary Secretary, before October 20th.

Stirling District Asylum, Larbert.—Second Medical Officer. Salary £80 per annum, with board and lodging. Applications and testimonials to the Medical Superintendent before October 17th.

West Riding Asylum, Wakefield.—Two Resident Clinical Assistants. Appointment tenable for six months. No salary, board and furnished apartments provided. Applications to the Medical Superintendent at once.

**Non-Resident Medical Officers.**

St. Olave Board of Works.—Medical Officer of Health for the District; not less than 25 or more than 45 years of age; must reside in or within one mile of the district. Salary £200 per annum. Applications to Edric Bailey, Clerk, Vine Street, Tooley Street, S.E., by October 15th.

**CHARTS**

PRICES (Post or Carriage Paid)—
1,000, 25s.; 500, 13s. 6d.; 250, 7s. 6d.; 100, 3s. 6d.; 50, 2s.; 25, 1s. 3d.

**TEMPERATURE, NURSING & DIET CHARTS,**

As used in almost all our principal Hospitals.

THE BEST AND CHEAPEST PUBLISHED.

**WODDERSPOON & CO.,**
7, SERLE ST., LINCOLN'S INN, W.C.

